# The role of domestic violence in fatal mass shootings in the United States, 2014–2019

**DOI:** 10.1186/s40621-021-00330-0

**Published:** 2021-05-31

**Authors:** Lisa B. Geller, Marisa Booty, Cassandra K. Crifasi

**Affiliations:** 1 Educational Fund to Stop Gun Violence, 805 15th St. NW, Washington, DC, 20005 USA; 2 Department of Health Policy and Management, Johns Hopkins Center for Gun Violence Prevention and Policy, Johns Hopkins Bloomberg School of Public Health, Baltimore, MD USA

**Keywords:** Domestic violence, Firearms, Mass shootings, Intimate partner violence

## Abstract

**Background:**

Fatal mass shootings, defined as four or more people killed by gunfire, excluding the perpetrator, account for a small percentage of firearm homicide fatalities. Research has not extensively focused on the role of domestic violence (DV) in mass shootings in the United States. This study explores the role of DV in mass shootings in the United States.

**Methods:**

Using 2014–2019 mass shooting data from the Gun Violence Archive, we indexed our data by year and mass shooting and collected the number of deaths and injuries. We reviewed news articles for each mass shooting to determine if it was 1) DV-related (i.e., at least one victim of a mass shooting was a dating partner or family member of the perpetrator); 2) history of DV (i.e., the perpetrator had a history of DV but the mass shooting was not directed toward partners or family members); or 3) non-DV-related (i.e., the victims were not partners or family members, nor was there mention of the perpetrator having a history of DV). We conducted descriptive analyses to summarize the percent of mass shootings that were DV-related, history of DV, or non-DV-related, and analyzed how many perpetrators died during the incidents. We conducted one-way ANOVA to examine whether there were differences in the average number of injuries or fatalities or the case fatality rates (CFR) between the three categories. One outlier and 17 cases with unknown perpetrators were excluded from our main analysis.

**Results:**

We found that 59.1% of mass shootings between 2014 and 2019 were DV-related and in 68.2% of mass shootings, the perpetrator either killed at least one partner or family member or had a history of DV. We found significant differences in the average number of injuries and fatalities between DV and history of DV shootings and a higher average case fatality rate associated with DV-related mass shootings (83.7%) than non-DV-related (63.1%) or history of DV mass shootings (53.8%). Fifty-five perpetrators died during the shootings; 39 (70.9%) died by firearm suicide, 15 (27.3%) were killed by police, and 1 (1.8%) died from an intentional overdose.

**Conclusions:**

Most mass shootings are related to DV. DV-related shootings had higher CFR than those unrelated to DV. Given these findings, restricting access to guns by perpetrators of DV may affect the occurrence of mass shootings and associated casualties.

**Supplementary Information:**

The online version contains supplementary material available at 10.1186/s40621-021-00330-0.

## Background

Mass shooting fatalities account for a small percentage (1%) of firearm homicide fatalities in the United States, but they receive a substantial amount of media attention and may drive political discourse on gun violence (Gun Violence Archive n.d.-a; Centers for Disease Control and Prevention, National Center for Health Statistics n.d.). In the wake of a mass shooting, people seek to understand why the incident occurred and how similar incidents could be prevented in the future. Risk factors for various forms of gun violence — including community gun violence and suicide — are well-known but, given the rarity of mass shootings, less information is known about why people carry out mass acts of violence. Recent research points to domestic violence (DV) as a precipitating factor for many mass shootings (Zeoli and Paruk 2019; Webster et al. 2020). According to the Centers for Disease Control and Prevention (CDC), an intimate partner is anyone with whom a person has a close, personal relationship. Specifically, this could include “current or former spouses, boyfriends or girlfriends, dating partners, or sexual partners,” and can occur “between heterosexual or same-sex couples and does not require sexual intimacy” (Centers for Disease Control and Prevention 2018). The definition of DV, however, goes further, including not just intimate partners but also a person with whom the victim cohabitates or shares a child or family members (United States Department of Justice n.d.). For the purposes of this study, a fatal mass shooting was defined as four or more people killed by gunfire, not including the perpetrator.

Federal law prohibits purchase and possession of firearms for those who have been convicted of a misdemeanor crime of DV (Gun Control Act of 1968, 18 U.S.C. § 922(g)(9) 1968), yet misdemeanor crimes vary by state and some states do very little to prevent DV perpetrators from purchasing firearms nor do they take steps to remove guns from perpetrators who become prohibited. The relationship between DV and firearm violence is well established. Over half of all intimate partner homicides (IPH) are by firearm (Fox and Fridel 2017; Zeoli 2018). While firearms are used in intimate relationships to kill, they are also used to threaten and intimidate. Around 4.5 million women in the U.S. have been threatened with a firearm, and nearly 1 million women have been shot or shot at by an intimate partner (Sorenson and Schut 2018). When an abuser has access to firearms, the risk the female partner will be killed increases by 400% (Campbell et al. 2003). Risk for homicide is also elevated when a woman attempts to, or successfully does, leave her abusive partner (Campbell et al. 2003).

There is limited research on the role of DV in mass shootings and multiple victim homicides. Zeoli and Paruk (2019) analyzed mass shooters from 2014 to 2017 to assess 1) whether offenders had known histories of perpetrating DV or were suspected to have committed DV before the mass shooting, 2) were legal firearm purchasers, or 3) had been previously engaged in the criminal justice system in a way that would have resulted in a restriction on firearm purchase/possession. They found that 31.5% of mass shooters in their study had histories of perpetrating DV. Further, the authors found that mass shootings could be prevented if DV cases are known in the criminal justice system or offenders are prohibited from having guns under a domestic violence protective order (DVPO) and the law is effectively enforced. Zeoli and Paruk (2019) found that there were, on average, more fatal victims in cases where there was a mention of DV (average of 7.1 individuals killed) compared to where there was no mention of DV (average of 6.2 individuals killed). Their paper highlights the myriad of gaps in the system and potential for would-be mass shooters with a history of DV to fall through the cracks when laws are poorly implemented, leaving them capable of purchasing and possessing firearms.

Kivisto and Porter (2020) found that the use of a firearm in a domestic homicide (where the victims are either intimate partners or family members) increases the risk that there will be multiple fatalities, which was not the case when a firearm was used in a nondomestic homicide. When a male used a firearm in a domestic homicide, he was almost twice as likely to kill at least one other person compared to a male who did not use a firearm (Kivisto and Porter 2020). Furthermore, 4.6% of the domestic homicides in Kivisto and Porter’s (2020) study had more than one victim, compared to 3.3% of non-domestic homicides, meaning that there was an increased incidence of multiple victims in domestic homicides compared to nondomestic homicides.

It is not uncommon for IPH events to result in multiple victims, including perpetrator suicide and the death of family, friends, new dating partners of the victim, coworkers, children of the victim or perpetrator, strangers, or police officers (Zeoli 2018). Research shows that around 40% of male-perpetrated IPHs result in multiple fatalities, either with the perpetrator dying by suicide or additional homicides (Kivisto 2015). A study of IPH events in 16 states from 2003 to 2009 found that nearly 30% of IPV-related incidents resulted in multiple deaths, with a median of 2 deaths per incident and a range of 2 to 7 deaths (Smith et al. 2014). Nearly 50% of the additional deaths were children or other family of the abused intimate partner, 27% of the additional deaths were new intimate partners of the targeted partner, 20% were friends and acquaintances of the intimate partner, 3% were strangers, and 1% were law enforcement officers who were summoned to the scene (Smith et al. 2014).

Using the FBI’s Supplementary Homicide Report (SHR) from 1999 to 2014 and a definition of four or more shot and killed, Reeping et al. (2019) classified mass shooting data by whether shootings were DV-related or not. They found that, during their study period, 23.5% of the mass shootings were related to DV. It’s important to note that Florida data were excluded from their study because of nonparticipation in the FBI’s reporting system. In addition, a main limitation of the FBI’s definition of DV-related shootings is that it is driven by the relationship between the offender and the first victim, which could result in misclassification of a mass shooting if the intimate or domestic partner was not the first victim killed (Reeping et al. 2019).

There has been limited research focused on the role of DV in mass shootings or on the differences in case fatality rates (CFR) between mass shootings that are DV-related, history of DV, or non-DV related. In this study, we explored whether there was a correlation between DV and mass shootings and whether there were differences in the average number of injuries and fatalities for mass shootings that were DV, history of DV, or non-DV-related using data from the Gun Violence Archive (GVA).

## Methods

### Definition

As there is no legal definition of a “mass shooting” in the United States, disagreements exist over how best to operationalize the concept. However, the scholarly literature commonly defines mass shootings as shootings that result in four or more deaths by gunfire, excluding the perpetrator (Booty et al. 2019; Zeoli and Paruk 2019). For the purposes of this study, this is the definition of a fatal mass shooting that is used. The use of varying definitions results in different numbers of mass shootings being captured by different databases and may have affected the results of this study. For example, a 2019 analysis of five mass shooting databases found that there was little overlap in the number of shootings found across the five sources due to differences in definitions (Booty et al. 2019). While recent work has called for an expanded definition of mass shooting to include both fatal and non-fatal injuries, this work provides important information about the relationship between DV and mass shootings with four or more fatalities by gunfire, excluding the perpetrator (Booty et al. 2019).

### Data and measures

For this analysis, we reviewed GVA data on mass shootings from 2014 to 2019. The GVA began collecting information about shootings in the United States in 2014, and the database tracks the date of the incident, city, state, and address of the incident, number killed, and number injured. The GVA defines a mass shooting as, “[Four] or more shot and/or killed in a single event [incident], at the same general time and location not including the shooter” (Gun Violence Archive n.d.-a, n.d.-b). However, as our focus was on mass shootings with four or more fatalities by gunfire, not including the perpetrator, we applied our definition to the GVA data. This resulted in a sample size of 128 mass shootings across the study period, with an average of 21.5 mass shootings per year (Gun Violence Archive n.d.-a).

We indexed our data by year and mass shooting and collected the number of deaths and injuries. Two authors independently reviewed news articles on each mass shooting and categorized whether it was DV-related (i.e., at least one victim of a mass shooting was a dating partner or family member of the perpetrator); 2) history of DV (i.e., the perpetrator had a history of DV but the mass shooting was not directed toward partners or family members); or 3) non-DV-related (i.e., the victims were not partners or family members, nor was there mention of the perpetrator having a history of DV). If there was discrepancy between the two authors in how an incident was coded, the case was discussed with the PI and the researchers came to a consensus. Of the 128 mass shootings, 120 incidents (94.0%) were coded exactly the same way by both coders. In the eight remaining mass shootings (6.0%), both coders met with the PI and a consensus was easily reached in all eight cases. While the 3/22/2017 shooting could have been coded as a history of DV mass shooting because the victims of the shooting did not include family or partners of the shooter, we have chosen to code it as a DV-related mass shooting because the perpetrator specifically targeted and intended to kill his wife.

Using a similar methodology outlined in Zeoli and Paruk’s (2019) paper, we applied our definition of a mass shooting to the data in GVA and reviewed each shooting entry and the articles listed on GVA. In addition to providing articles, GVA codes shootings based on several characteristics, one of them being domestic violence. However, understanding that GVA may omit articles, or information regarding a given shooting may change as stories develop, we did a comprehensive Google search of articles relating to each shooting. Search terms used included the offender’s name, the date of the shooting, the location of the shooting, as well as the words “domestic violence” to identify any mentions of domestic violence. For the higher-profile mass shootings, there were often dozens of news articles, including many articles in national news outlets that tended to have thorough information about the offender and the victims of the mass shooting. For lower-profile shootings, we reviewed the top 5–10 news stories, which often came from local news sources, to identify if there were media mentions of either a history of DV or if the victims of the shooting were family or intimate partners of the offender.

If the news articles mentioned that the victims were current or former intimate partners or other family members, we coded that shooting as “DV-related.” An “intimate partner” is a current or former spouse, dating partner, someone whom the offender had a child in common or lived with. A “family member” is someone related to the offender (either by blood, like a sister, brother, or cousin, or through the intimate partner, such as a mother-in-law) but who does not fall under the “intimate partner” category. If at least one news article mentioned that the offender had a known history of domestic violence (which could include a current or former partner mentioning that he or she was abusive), but the victims of the mass shooting were not intimate partners or family members, those cases were coded as a “history of DV” shooting. Actions falling under the “history of DV” category include violence (physical or otherwise) or threats of violence against a current or former intimate partner or family member (as defined above). When neither DV nor a history of DV was mentioned in any news stories, we classified the shooting as “non-DV related.”

Following the methodology used in Zeoli and Paruk (2019), if any victims of shootings with multiple perpetrators were family and/or intimate partners of the perpetrator, the mass shooting was classified as DV-related. If at least one of the perpetrators for shootings with multiple perpetrators had a history of DV, it was classified as a history of DV shooting. All other shootings were classified as non-DV related. There were 17 cases where the perpetrator was unknown, and these cases were removed from our main analysis. It is possible that there was a bias in our results based on how these unknown cases were classified.

During our preliminary analysis, we assessed the data for potential outliers in the total victim, victim death, and victim non-fatal injury counts; the Pulse Nightclub shooting in 2016 and the Las Vegas shooting in 2017 were of particular concern. We identified the Las Vegas shooting as an outlier as there were 471 total victims which was greater than three standard deviations from the mean (139 total victims). However, Pulse only exceeded three standard deviations from the mean for victim deaths, so it remained in the main analysis. A secondary analysis including the Las Vegas shooting in the analysis is available as Supplemental Materials (see Supplemental Tables 1–4).

### Analytic methods

We conducted descriptive analyses to summarize the percent of mass shootings that were DV-related, history of DV, or non-DV-related. We conducted one-way ANOVA to examine whether there were differences in the average number of injuries or fatalities or the CFR between DV, history of DV, and non-DV-related mass shootings. We calculated the CFRs by category to reflect the total number killed over the total number injured and killed. We then calculated 95% confidence intervals for each CFR; category CFRs were determined to be significantly different at the *p* = 0.05 level if the 95% confidence intervals did not overlap. We analyzed how many perpetrators died during the mass shootings and noted whether they died by suicide or were killed by police. Finally, we created a “hybrid” category that combined DV-related shootings with history of DV shootings. A two-sample t-test was then conducted to determine whether this new hybrid DV-category had significantly different average victim fatalities and injuries from the non-DV-related shootings.

Analyses were conducted using Stata version 16.1 (StataCorp 2019). Institutional Review Board approval was not required for this non-human subjects review of publicly available data.

## Results

There were 128 mass shootings between January 1, 2014 and December 31, 2019. However, after removing the shootings where the perpetrator was unknown and after excluding the Las Vegas shooting as an outlier, we were left with 110 mass shootings in our study. These shootings resulted in 651 deaths, not including the perpetrators, and 283 non-fatal injuries. In 65 of the 110 shootings (59.1%) analyzed, at least one fatal or non-fatal victim was a partner or family member (Table 1). In 10 of the 110 shootings (9.1%), the perpetrator had a history of DV, but none of the victims of the 10 mass shootings were partners or family members. The remaining 35 mass shootings (31.8%) were non-DV-related (Table 1). Twelve of the mass shootings had multiple perpetrators. Of those 12 incidents, seven were non-DV-related, three were history of DV mass shootings, and two were DV-related mass shootings (results not shown). Eight of the mass shootings involved female perpetrators, with one of the eight shootings having two female perpetrators. Of those eight incidents, five were DV-related mass shootings, two were non-DV-related, and one was a history of DV mass shooting (although it involved two shooters and it was the male counterpart who had the history of DV, with the female having no known history of DV herself).

**Table 1:** Number of Mass Shootings by Year and by DV Category

	2014	2015	2016	2017	2018	2019	Total
**Number of mass shootings**	11	19	20	15	18	27	110
**Number of DV-related mass shootings**	9	11	10	11	10	14	65
**Number of history of DV mass shootings**	0	1	3	1	1	4	10
**Number of non-DV-related mass shootings**	2	7	7	3	7	9	35

Fifty-five perpetrators of 53 mass shootings died during the incident; 39 (70.9%) died by firearm suicide, 15 (27.3%) were killed by police, and one (1.8%) died from an intentional overdose. Of the 39 mass shooting perpetrators who died by firearm suicide, 36 (92.3%) were perpetrators of DV-related mass shootings and three (7.7%) were perpetrators of non-DV mass shootings. Forty-two of the 65 perpetrators of DV-related mass shootings (64.6%) died during the incident, with 36 of the 42 perpetrators (85.7%) dying by firearm suicide. Of the 15 perpetrators who were killed by police, five (33.3%) were perpetrators of DV-related mass shootings, four (26.7%) were mass shooting perpetrators with histories of DV, and six (40.0%) were perpetrators of non-DV mass shootings. The remaining perpetrator who intentionally overdosed in the aftermath of the mass shooting was a perpetrator of a DV-related mass shooting (results not shown).

On average, there were 5.0 fatal injuries and 1.0 non-fatal injury for DV-related shootings. Perpetrators with a history of DV killed an average of 10.5 individuals and non-fatally injured 9.0 people. For non-DV-related mass shootings, there were an average of 6.3 fatalities and 3.7 non-fatal injuries (Table 2). There were statistically significant differences between the average number of fatalities, non-fatal firearm injuries, and total (fatal and non-fatal) injuries for DV-related and history of DV mass shootings. The difference between the average number of fatalities, non-fatal firearm injuries, and total injuries for history and non-DV related mass shootings approached significance. The CFR for DV mass shootings was 83.7%, compared to 53.8% for history of DV and 63.1% for non-DV-related mass shootings (Table 2). The CFR for the DV-related mass shootings were significantly different from both the history of DV and non-DV-related mass shootings.

**Table 2:** Average Mass Shooting Victims by DV Category

	DV-related	History of DV	Non-DV-related
Average fatalities per shooting (SD)*	5.0 (2.9)	10.5 (14.1)	6.3 (4.0)
Average non-fatal injuries per shooting (SD)*	1.0 (3.5)	9.0 (16.3)	3.7 (6.9)
Average total (fatal and non-fatal) victims (SD)*	6.0 (6.0)	19.5 (30.3)	10.1 (10.1)
**Case Fatality Rate [95% CI]**	83.7% [74.9, 93.4]	53.8% [44.0, 65.2]	63.1% [55.0, 71.9]
**Total (fatal and non-fatal) victims**	387	195	352

Non-DV related is the reference group for significance

* denotes significance at *p* < 0.05

In 75 of the 110 (68.2%) shootings analyzed, at least one fatal or non-fatal victim was a partner or family member of the perpetrator or the perpetrator had a history of DV (Table 3). Perpetrators of either DV or history of DV mass shootings killed an average of 5.7 people and non-fatally injured an average of 2.0 individuals. The CFR for this hybrid DV category was 73.7% compared to 63.1% for non-DV-related mass shootings (Table 4).

**Table 3:** Number of Mass Shootings by Year (Hybrid DV and Non-DV categories)

	2014	2015	2016	2017	2018	2019	Total
**Number of mass shootings**	11	19	20	15	18	27	110
**Number of DV and history of DV mass shootings**	9	12	13	12	11	18	75
**Number of non-DV-related mass shootings**	2	7	7	3	7	9	35

**Table 4:** Average Mass Shooting Victims: Hybrid DV and Non-DV Related

	Hybrid DV-related	Non-DV-related
**Average fatalities per shooting (SD)**	5.7 (5.9)	6.3 (4.0)
**Average non-fatal injuries per shooting (SD)**	2.0 (7.1)	3.7 (6.9)
Average total (fatal and non-fatal) victims (SD)*	7.8 (12.8)	10.1 (10.1)
**Case Fatality Rate [95% CI]**	73.7% [66.9, 81.0]	63.1% [55.0, 71.9]
**Total (fatal and non-fatal) victims**	582	352

Non-DV related is the reference group for significance

* denotes significance at *p* < 0.05

## Discussion

This study provides insight into the role of DV in mass shootings in the U.S. and the lethality of such events. Between 2014 and 2019, in 68.2% of mass shootings, the perpetrator either shot or killed at least one partner or family member or had a history of DV. The CFR for DV-related mass shootings was 83.7%; put another way, only 16.3% of victims in DV-related mass shootings survived the incident compared to 46.2% of victims where the offender had a history of DV and 36.9% of victims in non-DV-related mass shootings. The CFR for the hybrid DV category was 73.7%. We found that DV-related mass shootings resulted in a 32.6% increase in the CFR when compared to non-DV related mass shootings. Using a hybrid CFR, we found that the hybrid DV-related and history of DV mass shootings resulted in a 16.8% increase in the CFR compared to non-DV related mass shootings. To our knowledge, this is the first paper to assess whether there are differences in CFR for mass shootings based on whether there was a connection to DV.

There are several potential explanations for why DV-related mass shootings have a higher CFR than incidents where the victims were not partners or family members. The intent behind a perpetrator who kills a partner or a member of his or her family may differ from someone who kills people seemingly indiscriminately. This may result in a greater intent to make sure all victims in a DV-related mass shooting are killed (Zeoli 2018). The motive behind a DV-related mass shooting may be revenge, jealousy, a desire to assert power and control, divorce, financial problems, or even suicidality (Auchter 2010; Kelley 2009; Zeoli 2018). Given the intent of the perpetrator, DV-related mass shootings may be more targeted than non-DV-related mass shootings, which could increase likelihood that the victims involved would be killed.

For non-DV-related mass shootings, the intent may be less clear. An article in the *National Institute of Justice Journal* explains that, for mass shootings, the “underlying motive sometimes appears to be unknown. Typically, mass shootings occur in a public place, with a single shooter, and most victims are killed or wounded indiscriminately” (Lopez et al. 2020). For some of the deadliest mass shootings in recent history, like the Tree of Life Synagogue shooting (2018) and the El Paso Walmart shooting (2019), the motive driving these shootings was likely related to religion or race/ethnicity. These shootings did not target a specific person, as in a DV-related mass shooting, but rather targeted a specific group of people. The potentially unclear motive and/or indiscriminate shooting may be one explanation for why, on average, fewer victims of non-DV-related mass shootings died from their wounds. Indeed, there are likely a number of factors that could explain this that were not controlled for in the current study, including type of firearm used, location and density of the mass shooting venue, location of wounds, and emergency services and law enforcement response time. Future research should seek to further understand why DV-related mass shootings appear to have a higher CFR than other mass shootings.

This paper highlights the importance of including both “public” and “private” mass shootings in discussions around preventing these incidents. By only focusing on “public” mass shootings, many DV-related mass shootings may be left out of the discussion. This oversight may lead to an assumption that most mass shootings occur at random, leading to missed opportunities for intervention, either through policies or programs, that could help reduce the burden of mass shootings. The results of this paper, that most mass shootings are related to domestic violence, highlights the need to focus on mass shootings more broadly.

Prior research has found that restricting access to guns by perpetrators of DV reduces IPH. Civil domestic violence protective orders (DVPOs) that cover dating partners (13%), prohibit firearm possession for temporary orders (13%), or require firearm relinquishment (12%) are all associated with reductions in IPH (Zeoli et al. 2018). However, effective enforcement of these laws is key to ensure that those prohibited because of a DVPO cannot obtain guns. Additionally, some individuals at risk for interpersonal violence (including mass shootings) or self-harm may not be prohibited from purchasing or possessing firearms. To address elevated risk among individuals, 19 states and DC have passed extreme risk protection orders (ERPOs), an evidence-based mechanism to temporarily remove firearms from individuals who are a threat to themselves or others (Bloomberg American Health Initiative n.d.). This study shows that most perpetrators of DV-related mass shootings died by suicide, highlighting that DV-related mass shooting perpetrators may be at an elevated risk for suicide. ERPOs are a promising tool that could be used to prevent suicides, and recent data shows that these laws have also been used in efforts to prevent mass shootings in California (Bloomberg American Health Initiative n.d.; Wintemute et al. 2019). However, ERPOs are a relatively new policy; future research should further explore the association between ERPOs and mass shootings and their potential impact on DV-related mass shootings in particular.

There are two main distinctions between this paper and Zeoli and Paruk’s paper. First, as noted above, Zeoli and Paruk (2019) found that the average number of fatal victims was higher for cases where there was a mention of DV. We found the opposite. While the case fatality rate in our paper was higher for DV mass shootings, there were more fatal victims, on average, for non-DV mass shootings. Second, Zeoli and Paruk (2019) found that 31.5% of the shooters in their study had histories of domestic violence. By creating a hybrid category that included both DV-related and history of DV cases, we found that in 68.2% of mass shootings between 2014 and 2019, the perpetrator either killed a family member or intimate partner in the mass shooting incident or had a history of DV. The current paper’s findings show that the vast majority of mass shootings in the United States are related to domestic violence and while, on average, DV-related mass shootings result in fewer fatalities, fewer victims of DV-related mass shootings survive compared to victims of non-DV related mass shootings.

This study has several limitations. This is a cross-sectional study that examines associations and cannot be used to assess causality. The GVA relies primarily on news reports to build its database. As a result, cases that do not receive media coverage, or do not show up in the other sources they pull from (e.g., local and state police reports), are unlikely to be captured by this database. This is likely to result in an undercounting of the true incidence of mass shootings in the U.S. However, GVA links to detailed information that may not be available in other datasets which allows for a richer analysis of the data. The relationship between the perpetrator of a mass shooting and the victims was not always known which could have introduced misclassification into our data. Further, we were unable to analyze cases where the perpetrator was unknown. Because of this limitation in the data, there is potential for measurement error that could have biased our findings. In addition, GVA updates data in real time and, as a result, there may be victims of mass shootings who did not die immediately and therefore were not recorded in the original death count of the shooting. Changes in the number of mass shooting deaths could affect how a mass shooting was classified for the purposes of this study. This paper did not explore whether the location of a mass shooting differed for DV compared to non-DV shootings. Future work should focus on differences in the location of shootings that are DV-related versus those that are not DV-related. The CFRs should be interpreted with caution because the definition of a mass shooting was restricted toward those where four or more people were killed, potentially inflating the CFRs. Future research should explore differences in CFRs across categories using an expanded definition of mass shootings. We did not assess how state firearm policies may affect the number of mass shootings or the likelihood that a mass shooting was DV-related in a state. Future research should continue to examine the role that policies that disarm or otherwise restrict access to guns by perpetrators of intimate partner violence (IPV) or DV have in reducing or preventing mass shootings. Future research should explore the role of DV in more broadly defined mass shootings (i.e., with multiple casualties, either fatal or non-fatal) to assess whether the findings in the paper hold true.

## Conclusions

DV, whether directly related or through a perpetrator’s history, plays an important role in mass shootings in the United States. DV-related mass shootings were associated with fewer casualties but a higher CFR; fewer victims survived the injuries sustained in a mass shooting that was associated with DV, highlighting the lethality of these events. Increased focus should be placed on disarming and restricting access to guns by perpetrators of IPV and DV.

## Supplementary Information

**Additional file 1:** **Supplemental Table 1.** Number of Mass Shootings by Year and by DV Category - Las Vegas Included. **Supplemental Table 2.** Average Mass Shooting Victims by DV Category - Las Vegas Included. Non-DV related is the reference group for significance. **Supplemental Table 3.** Number of Mass Shootings by Year and by DV Category (“unknown” included with “non-DV related,” Las Vegas excluded). **Supplemental Table 4.** Average Mass Shooting Victims - (“unknown” included with “non-DV related,” Las Vegas excluded). Non-DV related is the reference group for significance. * denotes significance at *p* < 0.05.

**Additional file 2.**

## Data Availability

The data analyzed in this study are available at Gun Violence Archive, https://www.gunviolencearchive.org/.

## Abbreviations

DV: Domestic violence
CDC: Centers for Disease Control and Prevention
IPH: Intimate partner homicide
DVPO: Domestic violence protective order
FBI: Federal Bureau of Investigation
SHR: Supplementary Homicide Report
GVA: Gun Violence Archive
CFR: Case fatality rate
ERPO: Extreme risk protection order
IPV: Intimate partner violence
