# Supplementary material for: The role of domestic violence in fatal mass shootings in the United States, 2014–2019
Source: Inj Epidemiol. 2021 May 31;8:38. doi: 10.1186/s40621-021-00330-0 (PMC8165999; doi:10.1186/s40621-021-00330-0)
Supplement: Supplementary file 1 — Additional file 1: Supplemental Table 1. Number of Mass Shootings by Year and by DV Category - Las Vegas Included. Supplemental Table 2. Average Mass Shooting Victims by DV Category - Las Vegas Included. Non-DV related is the reference group for significance. Supplemental Table 3. Number of Mass Shootings by Year and by DV Category (“unknown” included with “non-DV related,” Las Vegas excluded). Supplemental Table 4. Average Mass Shooting Victims - (“unknown” included with “non-DV related,” Las Vegas excluded). Non-DV related is the reference group for significance. * denotes significance at p < 0.05. [file 40621_2021_330_MOESM1_ESM.docx]

**Supplemental table 1: Number of Mass Shootings by Year and by DV Category - Las Vegas Included**

|  | **2014** | **2015** | **2016** | **2017** | **2018** | **2019** | **Total** |
| --- | --- | --- | --- | --- | --- | --- | --- |
| **Number of mass shootings** | 11 | 19 | 20 | 16 | 18 | 27 | 111 |
| **Number of DV-related mass shootings** | 9 | 11 | 10 | 11 | 10 | 14 | 65 |
| **Number of history of DV mass shootings** | 0 | 1 | 3 | 1 | 1 | 4 | 10 |
| **Number of non-DV-related mass shootings** | 2 | 7 | 7 | 4 | 7 | 9 | 36 |

**Supplemental table 2: Average Mass Shooting Victims by DV Category - Las Vegas Included**

|  | **DV-related** | **History of DV** | **Non-DV-related** |
| --- | --- | --- | --- |
| **Average fatalities per shooting (SD)** | 5.0 (2.9) | 10.5 (14.1) | 7.8 (9.8) |
| **Average non-fatal injuries per shooting (SD)** | 1.0 (3.5) | 9.0 (16.3) | 15.0 (68.2) |
| **Average total (fatal and non-fatal) victims (SD)** | 6.0 (6.0) | 19.5 (30.3) | 22.9 (77.5) |
| **Case Fatality Rate [95% CI]** | 83.7% [74.9, 93.4] | 53.8% [44.0, 65.2] | 34.3% [30.4, 38.5] |
| **Total (fatal and non-fatal) victims** | 387 | 195 | 823 |

Non-DV related is the reference group for significance.

**Supplemental table 3: Number of Mass Shootings by Year and by DV Category (“unknown” included with “non-DV related,” Las Vegas excluded)**

|  | **2014** | **2015** | **2016** | **2017** | **2018** | **2019** | **Total** |
| --- | --- | --- | --- | --- | --- | --- | --- |
| **Number of mass shootings** | 13 | 22 | 24 | 18 | 19 | 31 | 127 |
| **Number of DV-related mass shootings** | 9 | 11 | 10 | 11 | 10 | 14 | 65 |
| **Number of history of DV mass shootings** | 0 | 1 | 3 | 1 | 1 | 4 | 10 |
| **Number of non-DV-related mass shootings** | 4 | 10 | 11 | 6 | 8 | 13 | 52 |

**Supplemental table 4: Average Mass Shooting Victims - (“unknown” included with “non-DV related,” Las Vegas excluded)**

|  | **DV-related** | **History of DV** | **Non-DV-related** |
| --- | --- | --- | --- |
| **Average fatalities per shooting (SD)** | 5.0 (2.9) | 10.5 (14.1)* | 5.7 (3.5) |
| **Average non-fatal injuries per shooting (SD)** | 1.0 (3.5) | 9.0 (16.3)* | 3.3 (6.3) |
| **Average total (fatal and non-fatal) victims (SD)** | 6.0 (6.0) | 19.5 (30.3)* | 9.0 (9.0) |
| **Case Fatality Rate [95% CI]** | 83.7% [74.9, 93.4]* | 53.8% [44.0, 65.2] | 63.7% [56.7, 71.4] |
| **Total (fatal and non-fatal) victims** | 387 | 195 | 466 |

Non-DV related is the reference group for significance.

* denotes significance at p < 0.05
